# Rural parents’ attitudes and beliefs on the COVID-19 pediatric vaccine: An explanatory study

**DOI:** 10.1371/journal.pone.0278611

**Published:** 2022-12-07

**Authors:** Rachael Lacy, Jini Puma, Michael Tubolino, David LaRocca, Lori A. Crane, Lisa Miller, Chad D. Morris, Sean T. O’Leary, Jenn A. Leiferman

**Affiliations:** 1 Rocky Mountain Prevention Research Center, Colorado School of Public Health, University of Colorado, Aurora, Colorado, United States of America; 2 Department of Community and Behavioral Health, Colorado School of Public Health, University of Colorado, Aurora, Colorado, United States of America; 3 Department of Epidemiology, Colorado School of Public Health, University of Colorado, Aurora, Colorado, United States of America; 4 Department of Psychiatry, University of Colorado, Aurora, Colorado, United States of America; 5 Department of Pediatrics, University of Colorado, Aurora, Colorado, United States of America; University of Ibadan, NIGERIA

## Abstract

The coronavirus disease 2019 (COVID-19) first came to the Unites States in January 2020. Though adult and pediatric vaccines became available to the public, vaccine uptake among youth and particularly younger children has been gradual. This explanatory study aimed to better understand parents’ attitudes and beliefs of the pediatric COVID-19 vaccine and the barriers and facilitators to vaccine uptake in a rural community through a brief, online demographic survey, and in-depth qualitative interviews. Forty-one in depth interviews were conducted with parents (31-English and 10-Spanish-speaking) residing in rural and frontier counties in Colorado between September 2021 and February 2022. Six emergent themes related to COVID-19 pediatric vaccine uptake were identified among the population. These themes spanned the three levels of influence in the Social Ecological Model (individual, interpersonal, and community levels). The six themes were identified as such; 1) Vaccine accessibility was associated with pediatric COVID vaccine uptake in rural communities, 2) Previous pediatric vaccine behaviors were not associated with COVID-19 pediatric vaccine uptake, 3) Perceived health status of a child or family member influenced pediatric COVID-19 vaccine uptake, 4) COVID-19 health seeking behaviors, like COVID pediatric vaccine uptake, are influenced by an individual’s prosocial or individualistic perspectives, 5) Child autonomy and “age of consent” frames vaccine decision making behaviors in parents, and lastly 6) Social networks impacted COVID-19 pediatric vaccine decision making. These findings inform next steps for COVID-19 pediatric vaccine uptake including targeted and tailored messaging for communities (cues to actions), engaging youth stakeholders, and identifying trusted sources to build rapport and trust between health professionals and community members. The growing vaccine hesitancy among parents has serious implications for disease eradication and future viral outbreaks. Understanding the perceived barriers and facilitators to pediatric vaccine uptake is important to maintain the health of our youth and communities.

## Introduction

The first case of coronavirus disease 2019 (COVID-19), the illness caused by the severe acute respiratory syndrome coronavirus 2 virus (SARS-CoV-2), was reported in the U.S. on January 20, 2020 [1]. Since then, over 50 million cases of COVID-19 have been confirmed in the U.S. [2] with a mortality rate of 287/100,000, exceeded only by a few countries [3]. COVID-19 disproportionately impacts certain ethnic and racial minority groups [4] and rural populations [5], with children in these communities as no exception.

On October 29, 2021, the United States Food and Drug Administration (FDA) authorized the emergency use of the Pfizer-BioNTech COVID-19 vaccine for children five years of age and older. Over seven million children have tested positive for SARS-CoV-2 [6] with this number growing daily. Despite this, as of December 2021, childhood COVID-19 vaccine uptake has been gradual, with 17% of children aged 5–11 receiving at least one dose of the two-dose COVID-19 vaccine [7]. In Colorado, there are nearly 2,000 approved vaccine providers in the states and as of February 22^nd^, 32% of children aged 5–11 are fully vaccinated against COVID-19, while nearly 61% of youth aged 12–17 are fully vaccinated [8]. These data, coupled with the fact that unvaccinated children 12–17 years of age are hospitalized at rates ten times higher than vaccinated youth [9] underscore the need to increase the uptake of pediatric COVID-19 vaccines. Further, a recent survey among urban parents suggests non-Hispanic black and Hispanic parents display higher levels of COVID-19 hesitancy compared to non-Hispanic white parents [10]. While there have been numerous surveys reported on parental COVID-19 vaccine attitudes overall, little is known about beliefs and behaviors among parents residing in rural communities. However, rural communities across the U.S. are vaccinating at a slower rate than their urban counterparts (67% compared to 79%, respectively) [11]. As such, this study aimed to better understand attitudes and beliefs regarding intent to vaccinate children against COVID-19 among parents and caregivers residing in rural, largely Hispanic communities in Colorado.

## Materials and methods

### Study design

This study utilized an explanatory study design, collecting both quantitative and qualitative data, with a larger emphasis on the qualitative findings. The quantitative component consisted of a brief survey with a demographic questionnaire and questions relating to adults’ attitudes and beliefs surrounding the COVID-19 vaccine and virus. Following the brief questionnaire, semi-structured qualitative interviews were conducted to further explore these phenomena.

### Participants and recruitment

Approval for this study was obtained from the Colorado Multiple Institutional Review Board (COMIRB # 21–3731). Participants were recruited from nine rural and frontier (fewer than six people per square mile) [12] southern counties in Colorado, between September 2021 and February 2022. Participants were eligible to participate if they were a parent (i.e., guardian who made medical decisions for a child aged 0–17), 18 years or older, and spoke English or Spanish. Participants were identified and recruited through existing partnerships with community-based organizations and local public health authorities, as well as grassroots efforts by community members and research liaisons. These existing relationships with community-based organizations were leveraged so that participants were assured of the study’s trustworthiness and credibility. Recruitment fliers in both English and Spanish were distributed throughout local health clinics, pharmacies, schools, libraries, faith-based and other community settings.

After completing an online consent form through Research Electronic Data Capture (REDCap) [13], eligible participants completed a brief questionnaire assessing age, race, ethnicity, household income, education level, marital status, political affiliation, zip code of primary residence, parity, age of child(ren), and COVID-19 vaccination status. The four item version of the Parent Attitudes about Childhood Vaccines (PACV-4) [14,15] instrument was also included to establish baseline parental vaccine hesitancy (i.e., “Have you ever delayed having your child get a shot for reasons other than illness or allergy?”, “I trust the information I receive about shots for my child”, “How concerned are you that a shot might not prevent a disease?”, “Overall, how hesitant about childhood shots would you consider yourself to be?”). The PACV-4 is a brief version of the PACV-15 that has demonstrated a sensitivity of 97% to 98% and a specificity of 79% to 81% for identifying vaccine hesitant parents [14,15].

Following completion of the brief survey, semi-structured qualitative interviews averaging 30-minutes in length were conducted in English or Spanish through a secure, online video platform. A semi-structured interview guide was developed using the Health Belief Model [16] (HBM) and the Social Ecological Model [17] (SEM) levels of intrapersonal, interpersonal and community influences. Interview questions explored parents’ and caregivers’ attitudes, beliefs, knowledge, and readiness regarding intent to vaccinate their child(ren) against COVID-19. Interviews (rather than focus groups) were chosen due to the sensitive nature of medical decision-making and the potentially polarizing conversations about COVID-19. The semi-structured interview guide included open-ended questions such as, “How ready, if at all, are you to get your child the COVID-19 vaccine?” which were then supplemented with follow ups such as “What would make you feel ready to vaccinate your child against COVID-19?” (See S1 File). The interview guide was also translated into Spanish prior to conducting the Spanish interviews.

English interviews were conducted by members of the study team trained in qualitative interviewing (RL, MT, or DL) with Spanish interviews conducted by Spanish-speaking, trained interviewers from the community. All Spanish speaking interviewers attended a qualitative interviewing training conducted by members of the research team. Interviews were audio-recorded, transcribed verbatim, translated (if conducted in Spanish), and stored on a password-protected hard drive. After completion of the interview, participants were compensated with a $20 electronic gift card.

### Trustworthiness of data

The qualitative portion of this study met the four criteria for trustworthiness of qualitative data: credibility, transferability, dependability, and confirmability [18]. Credibility was established during the creation of the semi-structured interview guide. Multiple investigators of the research team separately reviewed the interview guide to ensure the appropriateness of content. An interview protocol was developed before the interview process began and included participant consent, recording and transcribing of the interview verbatim, and storing the file on a password protected computer. While transferability was not the primary goal of this study due to the unique experiences of the population interviewed, clear assumptions were concluded about the population which may transfer to other rural, Hispanic populations. Purposive sampling was conducted to ensure voices of all community members were represented (e.g., Hispanic parents, female caregivers, male caregivers, and those with strong vaccine related opinions). In addition to conducting quality assurance protocols, dependability was met through an audit trail involving data collection, coding, and analyses. Finally, confirmability was met due to the triangulation of coding (two coders and one spot-coder) as well as the qualitative research training provided to all interviewers.

### Data analyses

Qualitative data analyses followed best practices for qualitative research and utilized data-driven and theory-informed approaches. Transcripts were reviewed multiple times to develop familiarity with the data, and then qualitative analysis of the transcripts was conducted using ATLAS.ti software. Constant comparison methodology was utilized to systematically reduce the data to codes [19]. Themes were then developed from the codes to describe parents’ and caregivers’ attitudes, beliefs, and knowledge regarding intent to vaccinate their child(ren) against COVID-19. The codebook used during analysis contained *a priori* codes related to the HBM and the SEM as well as inductive coding to explore and discover emergent themes outside of these two models’ constructs. Sampling decisions were guided by saturation that was reached at 41 interviews, after no novel themes or ideas emerged from participants in either the English or Spanish-speaking subgroups. Each transcript was coded separately by two research team members (RL and MT), and 15% of the transcripts were independently coded by a third researcher (DL) to establish consensus on coding structure and theme development. This third coder summarized themes and subthemes separate from the other two coders with the three coders then meeting to discuss themes and to reach consensus if there were differences in codes or emerged themes. To further explore these emergent themes, the interviews were grouped by vaccinated (76%) and unvaccinated (24%) and Spanish- (76%) and English-speaking (24%) parents. Sub analyses were conducted amongst these subgroups with an intention to compare and contrast the differences in these subgroups. All methodology was reviewed and approved by an investigative team trained in both quantitative and qualitative research methodologies, who were not involved in data collection or interview coding processes.

Questionnaire data was exported from REDCap, and all quantitative analyses were conducted using Statistical Analysis Software version 9.4. Descriptive statistics were run to understand the demographics of the sample, including age, gender, number of children, race, ethnicity, household income, political affiliation, education level, and COVID-19 vaccination status. Each response for the four PACV-4 items was scored as hesitant or non-hesitant, and parents were identified as vaccine hesitant if they endorsed a hesitant response for ≥2 items. This data was used to create a dichotomous PACV-4 variable, identifying parents as vaccine hesitant or non-vaccine hesitant. Due to the relatively small sample size, a Fisher’s exact test was used to examine the association between parental vaccine hesitancy via the PACV-4 and parental COVID-19 vaccination status.

## Results

### Demographics of sample

A total of 41 parents were interviewed (31 English-speaking and 10 Spanish-speaking) of which 38 (92.7%) were female with an average age of 38.5 years old. Similar to the ethnic breakdown of the rural community, 21 (51.2%) of our parents were Hispanic or Latino. Additionally, 33 (82.5%) reported an income less than $75,000, and over 36 (87.6%) had obtained a high school degree or higher. A Fisher’s exact test was used to examine the association between parent attitudes about childhood vaccines (PACV-4) and parental COVID-19 vaccination status with a significant association identified (p < 0.001), with 16 (39%) parents identifying as vaccine hesitant. Additional demographics are described in Table 1.

**Table 1:** Demographics of Interviewees.

Demographics	Total Sample N = 41 (%)	English Speaking n = 31 (%)	Spanish Speaking n = 10 (%)
Age			
Mean	38.5	39.7	34.9
Gender (Female)	38 (92.7)	29 (93.5)	9 (90.0)
Number of Children			
1	16 (39.0)	13 (41.9)	3 (30.0)
2	15 (36.6)	9 (29.0)	6 (60.0)
3	4 (9.8)	3 (9.7)	1 (10.0)
4 or more	6 (14.6)	6 (19.4)	0 (0.0)
Ethnicity			
Hispanic or Latino	21 (51.2)	11 (35.5)	10 (100.0)
Not Hispanic or Latino	18 (43.9)	18 (58.0)	0 (0.0)
Other	2 (4.9)	2 (6.5)	0 (0.0)
Race			
AIAN*	2 (4.9)	2 (6.5)	0 (0.0)
White	32 (78.0)	25 (80.5)	7 (70.0)
Other	2 (4.9)	2 (6.5)	0 (0.0)
Prefer not to answer	5 (12.2)	2 (6.5)	3 (30.0)
2020 Household Income			
<$24,999	14 (35.0)	8 (26.7)	6 (60.0)
$25,000-$74,999	19 (47.5)	16 (53.3)	3 (30.0)
>$75,000	7 (17.5)	6 (20.0)	1 (10.0)
Political Affiliation			
Republican	8 (20.5)	6 (20.0)	2 (20.0)
Democratic	18 (46.2)	14 (46.7)	4 (40.0)
Other	13 (33.3)	10 (33.3)	4 (40.0)
Education Level			
Less than high school	5 (12.3)	0 (0.0)	5 (50.0)
High school or equivalent	6 (14.6)	5 (16.1)	1 (10.0)
Some college/Associate degree	14 (34.1)	10 (32.3)	4 (40.0)
Bachelor’s degree or higher	16 (39.0)	16 (51.6)	0 (0.0)
COVID-19 Vaccination Status			
1 dose or more	33 (80.5)	23 (56.1)	10 (100.0)

*AIAN = American Indians and Alaska Natives.

**Fig 1:**
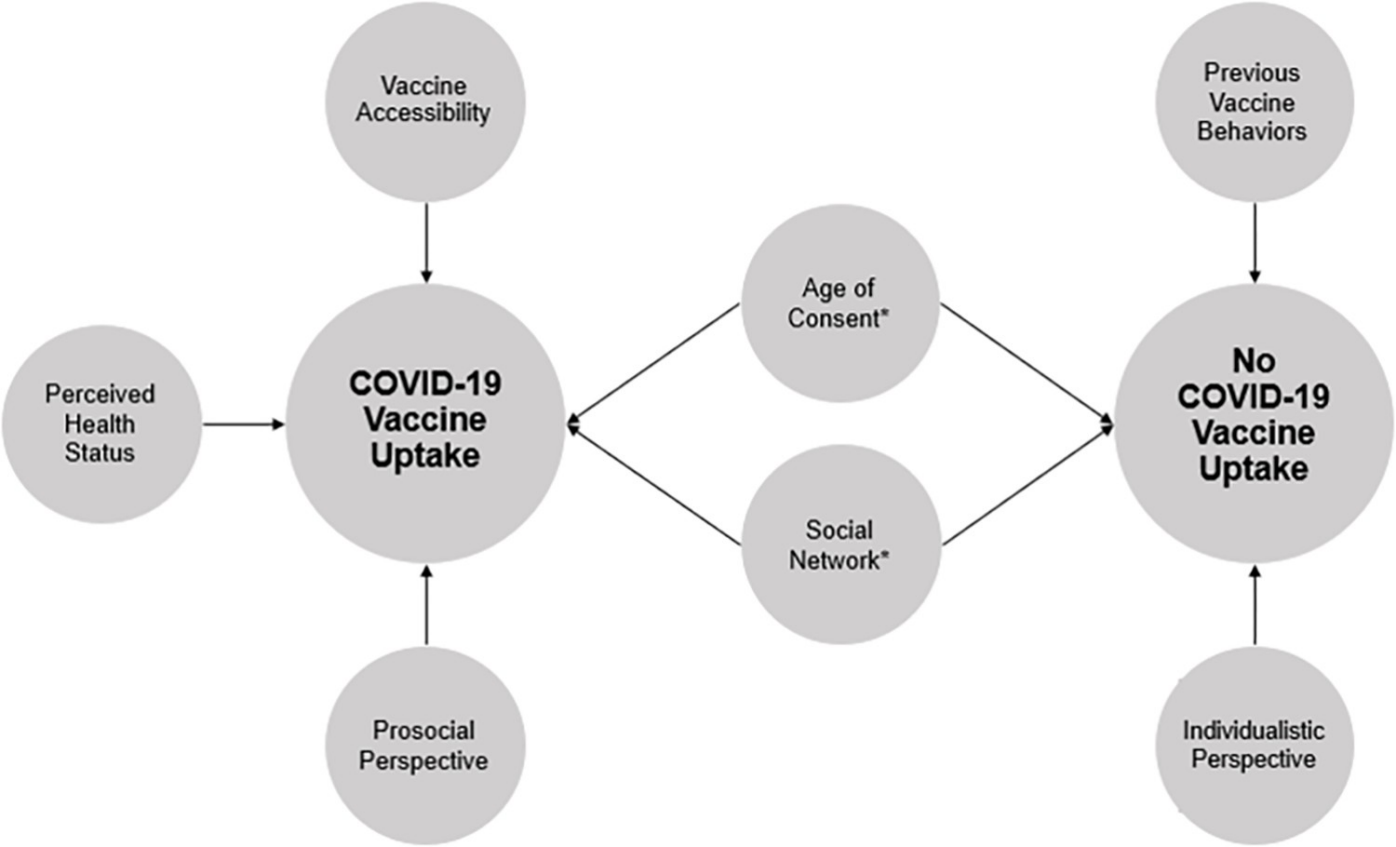
Emergent Themes Influence COVID-19 Pediatric Vaccine Uptake or No COVID-19 Vaccine Uptake. *Themes were influential in both vaccine uptake and non-vaccine uptake.

Cross-tabulations and findings presented in Tables 2 and 3. A total of six themes emerged from the qualitative data and are described below with direct quotations from participants shown in Table 4 and displayed in Fig 1.

**Table 2:** Cross-Tabulation of Parental COVID-19 Vaccination Status and PACV-4 Score.

COVID-19 Vaccination Status	Non-Hesitant PACV-4 Score	Hesitant PACV-4 Score
Unvaccinated	0 (0.0)	8 (19.5)
Vaccinated	25 (61.0)	8 (19.5)

A Fisher’s exact test demonstrated a statistically significant association (p < 0.001) COVID-19 vaccination status and PACV-4 score.

**Table 3:** Interviewee responses to the 4-Item Parent Attitudes About Childhood Vaccines (PACV-4) survey.

Item	Responses	n (%)
PACV-4 Items		
Have you received at least one dose of a COVID-19 vaccine?	Yes	33 (80.5)
	No	8 (19.5)
	I don’t know	0 (0.0)
Have you ever delaying having your child get a short for reasons other than illness or allergy?	Yes	5 (12.2)
	No	35 (85.4)
	I don’t know	1 (2.4)
I trust the information I receive about shot for my child.	Strongly agree	16 (39.0)
	Agree	16 (39.0)
	Not sure	8 (19.5)
	Disagree	1 (2.4)
	Strongly disagree	0 (0.0)
How concerned are you that a shot might not prevent a disease?	Not at all concerned	3 (7.3)
	Not too concerned	9 (22.0)
	Not sure	9 (22.0)
	Somewhat concerned	15 (36.6)
	Very concerned	5 (12.2)
Overall, how hesitant about childhood shots would you consider yourself to be?	Not at all hesitant	17 (41.5)
	Not too hesitant	14 (34.1)
	Not sure	3 (7.3)
	Somewhat hesitant	5 (12.2)
	Very hesitant	2 (4.9)
PACV-4 Score		
0–1 (non-hesitant)	-	25 (61.0)
2+ (hesitant)	-	16 (39.0)

**Table 4:** Thematic results and quotations.

Emergent Findings (Themes)	Supporting Quotations
**Vaccine accessibility was associated with pediatric COVID vaccine uptake in rural communities**	I just really feel like it’s available here…it’s really an self-initiative type of thing. I think, taking yourself or your children to go. There’s really nothing that prevents them locally here from getting it.
	Oh, yeah, there’s [vaccines] so many of them. So many different categories. It’s weird, Pfizer and Johnson & Johnson or something like that. There’s a lot here. There’s a lot available.
**Previous pediatric vaccine behaviors were not associated with COVID-19 pediatric vaccine uptake.**	I vaccinate for other things. You know, like I said, I’m not anti-vaccine by any means. It’s [COVID pediatric vaccine] just too soon.
	I’m not against vaccinating my kids, we have our immunizations up to date. However, this one, just, it’s hard for me to wrap my mind around and feel completely comfortable with.
**Perceived health status of a child or family member influenced pediatric COVID-19 vaccine uptake.**	I think it’s [pediatric vaccine] just another level of protection. You know? I think we feed our kids good food, we encourage our kids to get enough sleep, we get our kids outside, you know, we mentally stimulate them, we physically stimulate them. And this is one more piece of the puzzle that builds their ability to be resilient.
	I just don’t think it should be pushed on every person because as a healthy person who hasn’t had any complications their whole life, and is very active and exercises and is outside getting vitamin D every day. I don’t see the point [in the pediatric vaccine].
**COVID-19 health seeking behaviors, like COVID pediatric vaccine uptake, are influenced by an individual’s prosocial or individualistic perspectives.**	So it’s as important to protect us and protect others. It should be required for everybody who works in school to be vaccinated. That’s my point of view. So it’s part of like, be part of the solution at the same time, is it safe, I’m responsible and I work, you know, on a school, my responsibility is to get vaccinated to protect toddlers
	I don’t think that me doing something [pediatric vaccine] for someone else [is worth it]. Like putting something in my body that we have no idea what it’s going to actually do in 10 years
	We can’t change the fact that the virus is introduced to the world… So it all boils down to at the end of the day, individually, how you’re going to take care of that for yourself. So for example, myself, I’ve made a choice because I do not want to be vaccinated to practice the self-isolation you know, pretty much self-quarantine but for work for school, for myself, I’ve chose to stay home and practice being very cautious and aware of how I interact with others, my children, my family and, and keeping that regimen very strict.
**Child autonomy and “age of consent” frames vaccine decision making behaviors in parents.**	We’ve talked about it and she [the child] understands why I got the vaccine but she’s like I still don’t I still don’t want to and I’m like well like you can do your own research on whatever you want to do and figure it out from there
	Well, outside was the fact that she [the child] got, she got to make the choice [to vaccinate or not] … she got the opportunity to participate in an adult like type of decision making. So that was a big benefit, you know, and I know, it’s different being that she’s so close to 18 versus, you know, some younger [children]… was a big benefit to actually have that conversation with her as a pre adult.
	But I don’t know… it was one thing for me to decide for myself [to vaccinate or not]. . . It just feels like a really heavy burden to decide that for my daughter, who’s only 13 and has her whole life.
	I’ve always been like that… to me, it’s like, it’s your bod. So you [the child] get to have a say in it. Because I’m not going to sit there and I’m not going to hold you down and make you do something that you don’t want to do.
**Social networks impacted COVID-19 pediatric vaccine decision making.**	I tend to trust the medical community, especially locally, because we know them, like my kids’ Doctor, I went to high school with him.
	My sister’s in the medical field. So she had done a lot of research [regarding the vaccine]…my brother and sister in law, my husband. I think they all had gotten it before me. So I guess in my mind, that was like, proof that it [the vaccine] was okay. So that kind of eased my fears. And then my oldest son… he just knew that it was the right thing to do. He didn’t really have any questions about it. And then my daughter… she knew that her aunt had researched it, she felt comfortable with it. And so I mean, we did have conversations about well, what do you want to do? What do you think? And so that was really all that happened.

Footnote: At the time of interviews, eligible children were 5 and older.

### Themes

The HBM and SEM provided the guiding frameworks for our semi-structured interview guide. We were most interested in overlaying how rural parents utilized their interpersonal, intrapersonal, and community networks to make COVID-19 pediatric vaccine and virus decisions through the lens of the HBM’s constructs; perceived susceptibility, severity, benefits, barriers, cues to action, and self-efficacy.

#### Vaccine accessibility was associated with pediatric COVID vaccine uptake in rural communities

When initially rolling out FDA-approved vaccines for adults 18+, availability in rural Colorado was limited. At the time of these interviews, the availability of the vaccine was not cited as a significant barrier. However, perceived barriers related to access of the vaccine included: lack of knowledge regarding where and how to find a preferred vaccine brand, time off from work to account for common adverse side effects, time spent scheduling appointments and traveling to clinics, and distance from home/work to a vaccine location. Despite these barriers, high self-efficacy was perceived in vaccinated parents on obtaining a pediatric vaccination.

#### Previous pediatric vaccine behaviors were not associated with COVID-19 pediatric vaccine uptake

Numerous parents previously vaccinated their children on a routine childhood vaccine schedule but elected to not vaccinate against COVID-19. However, these individuals displayed higher perceived severity and viewed the COVID-19 vaccines as “different” or “riskier” than other established and well-known pediatric vaccines. Parents reported heightened concerns regarding the COVID-19 vaccines including the belief that vaccines can alter DNA, the vaccine came out “too fast”, uncertainty of the vaccine ingredients, mistrust of the vaccine effectiveness due to variants, and unknown short- and long-term outcomes of the vaccine.

#### Perceived health status of a child or family member influenced pediatric COVID-19 vaccine uptake

Confidence in the health of the family unit influenced vaccine uptake. Numerous parents held the belief that those who are healthy (e.g., no preexisting conditions, normal body mass index (BMI), participate in regular physical activity, eat healthy, and have a strong immune system) are less susceptible to SARS-CoV-2. The lack of perceived susceptibility due to lifestyle choices negatively influenced pediatric vaccine uptake. Parents also related their health status to a decreased likelihood of hospitalization or severe lasting impacts, suggesting low perceived severity, if infected with the virus and therefore viewed the vaccine unnecessary due to their or their child’s good health. Conversely, parents viewed the pediatric vaccine as an additional component in increasing their health confidence and overall health status.

#### COVID-19 health seeking behaviors, like COVID pediatric vaccine uptake, are influenced by an individual’s prosocial or individualistic perspectives

Parents fell on a spectrum from prosocial to individualist beliefs. Parents varied among their definitions of “health responsibility”. Several felt that to best protect themselves and their family, an individualistic approach to health was necessary while others demonstrated a prosocial view towards the health of others with voluntary behaviors intended to benefit the community (i.e., vaccinating themselves, vaccinating their children, social distancing, and mask wearing). Parents who held individualistic views regarding the COVID-19 pediatric vaccine displayed a heightened level of vaccine hesitancy and concerns. Conversely, parents with a prosocial attitude indicated higher readiness and likelihood to vaccinate their children once eligible.

#### Child autonomy and “age of consent” frames vaccine decision making behaviors in parents

Parents allowed children to influence vaccine behavior and decision-making. Children aged 12–17 were viewed as old enough to decide to vaccinate or not and to weigh the potential consequences of these decisions. Numerous parents perceived these conversations related to a child’s body autonomy as an important learning opportunity for the child. While these discussions were not the sole deciding factor in parents’ decision to vaccinate their children, this concept of childhood body autonomy heavily influenced decision making. Children aged 11 and younger were perceived as having fewer capabilities to make an informed decision for themselves. Many parents felt that until a child could fully consent, they were uncomfortable with vaccinating their child. These beliefs were highest among parents with children aged five or younger.

#### Social networks impacted COVID-19 pediatric vaccine decision making

Parents relied heavily on their social networks to inform vaccine decision-making and sought information from a variety of trusted sources. Parents brought their own perceived knowledge, attitudes, and skills to the decision-making process and leveraged their interpersonal, organizational, and community social networks. Most parents viewed their interpersonal social networks, such as personal connections with vaccinated healthcare workers, as a valuable and trusted information source. Parents described triggers to their decision making (cues to action) such as a family member or friend having a positive vaccination experience and/or a provider’s recommendation to vaccinate themselves of their children.

#### Subthemes

*Vaccinated and Unvaccinated Parents*. Notable differences existed between vaccinated and unvaccinated parents. Vaccinated parents spoke more frequently about their responsibility to protect their children, the value of science, and the benefits of vaccinations. Vaccinated parents often mentioned external cues to actions like provider’s recommendations or stories from personal healthcare connections of the severity of contracting the COVID-19 virus influencing their decision-making processes. Although these parents wanted more information surrounding childhood vaccines, vaccinated parents tended to trust the public health institutions such as the CDC, WHO, and were more inclined to seek trusted scientific journals. Additionally, vaccinated parents documented less perceived barriers to the COVID-19 pediatric vaccination and increase perceived benefits of the vaccine.

Unvaccinated parents were more likely to trust interpersonal connections in their decision making (e.g., partners, friends, and extended family members). Unvaccinated parents often cited frustrations with inconsistent messaging surrounding the COVID-19 vaccines and virus. These parents were highly focused on perceived associated risks of the vaccine (e.g., heart complications, fertility issues, and DNA modification) and downplayed the protective benefits of vaccines and described less perceived benefits than vaccinated parents.

*English speaking and spanish speaking parents*. Similarities between these groups involved perceived benefits of the vaccine, concerns regarding short and long-term effects of vaccines, and a need for more consistent and robust information regarding COVID-19 vaccine and the virus. The Spanish-speaking parents reported more trust in their local medical community and cited similar providers whom they felt trustworthy and knowledgeable than English-speaking parents. Spanish-speaking parents also requested a community forum with chosen health care professionals to discuss the associated risks and benefits of the pediatric vaccine. Spanish-speaking mothers also mentioned their trust in their husbands when making medical decisions and the importance of a community-centered approach for eliminating the virus.

## Discussion

### Individual level

There were multiple key themes which influenced pediatric vaccine uptake among our rural sample. Individual levels of influence were informed by parents’ perceived susceptibility based on their overall general health status. While a recent global survey, where almost half of American adults resided in a rural community, indicated that general health was not a significant predictor in COVID-19 vaccine uptake, [20] our respondents with good perceived health reported more hesitancy towards the pediatric COVID-19 vaccine. This belief of perceived susceptibility via health status can inform targeted messaging in communities where individualism are fundamental values. In previous vaccination work, tailored and targeted messages have been effective in behavior change [21]. A recent survey of adults living in California found that “nudging” and reminders based on a prosocial or personal framework were influential in COVID-19 vaccine uptake [22]. Tailored messaging targeting disease risks associated with contracting the virus as well as additional conferred economic and social benefits of containing disease spread [20] are needed to encourage uptake. The CDC recommends examples of these types of evidence-based messages rooted in cues to action including *“Vaccination boosts the body’s natural defenses against disease to keep you free of infection*” and “*Vaccination helps you take personal control of your life and allows you to be free to live a healthy life”* for communities rooted in liberty (27).

Additional individual influences on vaccine uptake centered on beliefs each parent held on the continuum from prosocial to individualism. Parents who described more individualistic beliefs reported that the governing bodies should not mandate the vaccine in school-aged children and each community member should “take ownership of their own health”. This finding is consistent with extant literature in which 82% of rural parents opposed a vaccine mandate for eligible students, compared to 57% of urban parents [23]. Moreover, another national survey suggests that six in ten rural residents, compared to less than half of urban and suburban, believe vaccination is a personal choice, while less than half believe it is a part of everyone’s responsibility to protect the health of the community [24]. Pediatric vaccine uptake in rural communities is conditional upon buy in from the community. The unvaccinated parents showed high distrust in larger government agencies and preferred personal health care connections when making decisions. Recent strategies to build vaccine confidence in unvaccinated individuals include vaccine ambassadors, motivational interviewing, effective messaging by trusted messengers (cue to action), provider recommendations (cue to action), and messaging to combat misinformation [25].

In addition to the individualist beliefs parents are bringing to the vaccine decision making process, parents in our study also encouraged their children to participate in the vaccine decision-making process. Particularly, our findings suggest parents with children aged 12–17 desire to engage their child in the healthcare decision-making process. Parents reported children refused the vaccine for various reasons (e.g., fear of pain at injection site and uncomfortable side effects). This shift of decision making responsibility may be in part due to omission bias, where parents consider the risks of vaccinating to outweigh the risks from vaccine refusal [26]. Many parents mentioned feeling absolved of blame for negative consequences (e.g., fear of DNA change, infertility, and death) relating to vaccinating or not. These perceived biases may be due from information circulating related to short- and long-term effects and even death from the COVID-19 vaccine. While this appears to be novel to the COVID-19 vaccine, targeted interventions empowering young people to speak up about their choice to vaccinate may enhance vaccine uptake. A recent study suggests a sliding scale of decision making among young people, allowing adolescents greater autonomy for medical decisions like vaccinations[27] and is consistent with our rural parents views surrounding their adolescents’ ability to make health related decisions. Additional evidence-based strategies suggested by UNICEF include inviting youth and adolescents to participate as stakeholders in vaccine conversations and strengthen partnerships among the community’s youth to amplify their views and opinions [28].

### Interpersonal level

In addition to the beliefs that each parent and child brings to the decision process, other influences are interacting. Our sample indicated a lack of perceived benefits of the pediatric vaccine (e.g., my child can still get sick with the vaccine) as well as lower perceived severity (e.g., COVID-19 is no more severe than a common cold), and perceived susceptibility (e.g., children are not at a risk, like adults, for contracting COVID-19). Rooted in these perceptions, many parents remain hesitant to vaccinate their children against COVID-19 despite 79% of children in the U.S. vaccinated with the combined 7-vaccine series by 24 months of age [29]. In part, parents still expressed distrust around the efficacy or safety of the COVID-19 vaccine [24] and a recent national survey of parents found that a little over half of parents’ state that they do not have enough information regarding COVID-19 vaccine effectiveness to feel confident in vaccinating their child [30].

Parents across the U.S. have expressed concerns related to vaccine safety and potential side effects of the pediatric COVID-19 vaccine, particularly for younger children [31]. A survey among rural communities showed parents were *“very”* or *“somewhat concerned”* regarding serious side effects of the vaccine (high perceived severity of the vaccine) [24] with 50% of rural American parents stating they will “*definitely not”* vaccinate their children 5–17 years of age [23]. Similar to our findings, a recent national survey among parents found that local and culturally appropriate pediatricians or health care providers were the most trusted information source regarding COVID-19 [32]. In a recent qualitative study exploring COVID-19 vaccine perceptions within Latino urban families, there was a strong desire for community-led advocacy and flow of information [33] with clear messaging regarding misconceptions. This is similar to the “call for action” by our rural Spanish-speaking parents who requested a community forum led by trusted health professional to address misinformation and foster trust among the community and health professionals. This previous survey also suggested schools, churches, Latino clinics, and supermarkets as a viable source for information dissemination [33]. Our Spanish-speaking population also mentioned cultural events and health fairs as trusted sources. This framing of information through a cultural lens is also applicable to the vaccinated and unvaccinated groups as information needs to be tailored to these communities by ingrained, trusted individuals.

Other trusted community experts were schools, particularly teachers and school nurses. In a nationwide survey of parents, schools that encouraged childhood vaccination saw increased vaccine uptake, particularly among children aged 12–17 [30]. Despite schools being a trusted source, only 36% of rural schools have reported encouraging parents to vaccinate their children [23]. Thus, rural public health agencies should continue to leverage communication efforts through the school system and foster personal relationships within their community to deliver timely messaging around the COVID-19 pediatric vaccine.

### Community level

Rural communities have historically been faced with barriers surrounding availability of healthcare services [34]. Although parents in our sample perceived few barriers and adequate availability of the COVID vaccine, many reported low self-efficacy alongside barriers associated with access and equity including unpaid time off from work, travel time to a clinic, vaccine appointment duration, time spent finding a qualified clinic, health literacy, and compromised trust in the health care system. In a national survey, parents with household incomes less than $50,000 voiced concerns similar to our population relating to vaccine access [30]. These consistent emerging concerns related to access to health care, particularly vaccinations, underscore the need to promote equitable access to vaccines. For example, in Southern Colorado, future solutions and strategies may include leveraging community events such as faith-based group meetings, county fairs, rodeos, and local cultural events like the celebration of “Dia de los Muertos” to reduce several of these diversity and equity barriers [35]. Such opportunities exist throughout the county and will need to be tailored to specific communities.

### Limitations

Limitations of this study include the different stages of federal vaccine approval throughout the interview timeframe. The opinions expressed by parents could have been impacted by FDA approval of the pediatric vaccine for their children’s ages, yet this is unavoidable due to the ever-changing landscape of the pandemic. Additionally, our sample included a higher number of vaccinated than unvaccinated parents, potentially suggesting more favorable opinions towards vaccinations, in general. While we aimed to have a sample representative of the population, the polarizing nature of COVID-19 created challenges in recruiting non-vaccinated individuals and 33 of the parents had received at least one dose of the COVID-19 vaccine at the time of the interview. Additionally, an oversampling of highly educated parents occurred with 84% of our sample reporting having at least some college or trade school, while only 25% of the rural population aged 25+ have completed some college [36]. Education levels have been positively correlated with vaccination rates in individuals and, therefore, may influence a parent’s decision to vaccinate themselves and their children. Finally, this study may not include a representative sample of all Hispanic or Latino parents in rural communities.

## Conclusions

The landscape of COVID-19 is constantly changing, with new COVID-19 variants emerging. Information and strategies to reach vaccine hesitant populations are critical. Ensuring that access to vaccines is equitable in low-resourced communities is an important step in increasing vaccination rates. Identifying trusted community partners to advocate and spread awareness of the benefits of vaccines, while inviting youth and unvaccinated individuals to partake in the conversations, could be effective in increasing vaccine uptake and uniting communities to address public health initiatives. COVID-19 has highlighted the need for increased relationship building within communities, particularly in rural areas where multi-generational families often reside. Generating buy-in of health initiatives among younger individuals could impact the amount of knowledge in familial units.

## Supporting information

S1 File Interview guide. Seme-structured interview guide used among interviewee participants.
(PDF)
