# Supplementary material for: Rural parents’ attitudes and beliefs on the COVID-19 pediatric vaccine: An explanatory study
Source: PLoS One. 2022 Dec 7;17(12):e0278611. doi: 10.1371/journal.pone.0278611 (PMC9728923; doi:10.1371/journal.pone.0278611)
Supplement: S1 File — Seme-structured interview guide used among interviewee participants. (PDF) [file pone.0278611.s001.pdf]

Protocol #: 21-3731
Project Title: RMPRC COVID-19 Supplement
Principal Investigator: Jenn Leiferman and Jini Puma
Version Date: 09/14/2021

Consent

1. What language would you like this interview to be conducted in?
    a) English
    b) Spanish
    c) No preference

Pre-survey

2. What is your age (in years)?
    a) _______

3. What sex were you assigned at birth, on your original birth certificate?
    a) 1, Male
    b) 2, Female
    c) 3, Other

4. How many children do you have under the age of 18 currently living in your household?
    a) 0
    b) 1
    c) 2
    d) 3
    e) 4
    f) 5
    g) 6+
        1. What are the ages of your children? Please select all that apply.
            a) 0-5
            b) 6-11
            c) 12-18

5. What is your ethnicity?
    1. Hispanic or Latino
    2. Not Hispanic or Latino
    3. Other (Please specify): ________________

6. What is your race? (Select all that apply)
    a) American Indian or Alaska Native
    b) Asian
    c) Black or African American
    d) Native Hawaiian or Other Pacific Islander
    e) White
    f) Other
    g) Prefer not to answer

7. Are you currently covered by any form of health insurance or health plan?
    a) Yes
    b) No
    c) I don't know

8. In 2020, what was your total household income before taxes?
    a) Less than $15,000
    b) $15,000-$19,999
    c) $20,000-$24,999
    d) $25,000-$34,999

e) $35,000-$49,999
f) $50,000-$74,999
g) $75,000-$99,999
h) $100,000 and above

9. What is the highest level of education you have completed?
    a) Less than high school
    b) High school or equivalent (e.g., GED)
    c) Some college, including associate degree or trade school
    d) Bachelor's degree or higher

10. What is your marital status?
    a) 1, Married
    b) 2, Divorced
    c) 3, Widowed
    d) 4, Separated
    e) 5, Never married
    f) 6, Member of unmarried couple

11. What political party affiliation do you most align with?
    a) 1, Republican Party
    b) 2, Democratic Party
    c) 3, Independent Party
    d) 4, Libertarian Party
    e) 5, Other

12. What zip code is your primary residence in?
    a) _ _ _ _ _

13. Have you already received your COVID-19 vaccine?
    a) Yes
    b) No
        1. How likely are you get a COVID-19 vaccine?
            a) Definitely get a vaccine
            b) Probably get a vaccine
            c) Not sure
            d) Probably not get a vaccine
            e) Definitely not get a vaccine
    c) I don't know

14. Have you ever delayed having your child get a shot for reasons other than illness or allergy (including the hepatitis B shot at birth)?
    a) Yes
    b) No
    c) Don't know

15. I trust the information I receive about shots for my child.
    a) Strongly Agree
    b) Agree
    c) Not sure
    d) Disagree
    e) Strongly disagree

16. How concerned are you that a shot might not prevent a disease?
    a) Not at all concerned
    b) Not too concerned
    c) Not sure
    d) Somewhat concerned
    e) Very concerned

17. Overall, how hesitant about childhood shots would you consider yourself to be?
    a) Not at all hesitant
    b) Not too hesitant
    c) Not sure
    d) Somewhat hesitant
    e) Very hesitant

Survey

Hello. Thank you for taking the time to talk to me and for agreeing to participate in this interview regarding your experiences around childhood vaccines.

I would like to start by introducing myself. My name is [name of interviewer] and I am with the Rocky Mountain Prevention Research Center. The purpose of this interview is to learn more about your experience as a parent choosing whether to vaccinate your child or children again the COVID-19 virus. We will be asking questions regarding routine childhood vaccines and specifically about the COVID-19 vaccine in both adults and children. I want to take a few minutes to tell you what to expect from our discussion today.

First, I want you to understand that your participation in this interview is completely voluntary. Our priority is your safety and well-being, so if at any time you feel the need to end the interview (e.g., you are having a challenging time answering questions), please let me know.

Second, I want to remind you that the discussion here is confidential. I will not report your comments by name, and I do hope that you feel able to be honest with your responses to the questions I ask. If you need me to clarify any questions, please let me know. Please note, that the purpose of recording is to accurately capture what you say.

Third, I want you to know that there are no right or wrong answers. I want to know your honest opinions regarding vaccines in children and adults.

This interview should only take us 20-30 minutes and you will receive a $20 gift card for participating in it.

Does that all sound ok?
Do you have any questions before we get started?
Is it okay with you if I begin recording now?

Introduction

1. This has been such an unusual last year and a half. Can you tell me how it has been for you?

2. Tell me about your experience with any COVID-19-related health concerns.
    a) Have you ever had COVID-19? Do you know anyone who had COVID-19? Tell me more about that.
    b) How easy do you think it is to catch COVID?
    c) How concerned are you about yourself or a family member getting COVID?
    d) How concerned are you about yourself or your family having a severe illness due to the COVID-19 virus?

e) Have you gotten the COVID-19 vaccine? Tell me about that decision.
f) Which age group or groups do you believe are most at risk for contracting COVID-19?
g) Thinking about your family and friends, how many of them have gotten vaccinated?
    1. Thinking about your immediate family, which of them have gotten vaccinated?
    2. Thinking about your extended family, which of them have gotten vaccinated?
    3. Thinking about your friends, which of them have gotten vaccinated?
h) What kinds of reasons have those people had for getting the vaccine or not getting it?
    I. Thinking about your immediate family, which of them have gotten vaccinated?
    II. Thinking about your extended family, which of them have gotten vaccinated?
    III. Thinking about your friends, which of them have gotten vaccinated?

3. Tell me about your child(ren).
    a) How many children do you have and how old are they?
    b) If they have multiple children, ask them to focus on the child aged 2-5 years old for the next few questions.

4. Tell me about your experience with any COVID-19 related health concerns for your child(ren).
    a) Has your child(ren) had COVID-19? Have any children at your child(ren)'s school had COVID?
    b) Do you think COVID-19 is a serious health concern for children? Tell me more about this.
    c) How concerned are you about your child getting COVID-19 once resuming in-person learning?
    d) How concerned are you about your child having a severe reaction to the COVID-19 vaccine once it is approved for their age group?
    e) How do your opinions differ regarding intent to vaccinate with your child aged xx AND xx?

5. How ready, if at all, are you to get your child the COVID-19 vaccine?
    a) What would make you feel ready to vaccinate your child?
    b) What do the people around you, your friends and family members, think about your child/ren getting the COVID vaccine? How important is it to you what they think about the COVID vaccine?
    c) Whose opinion is most important to you in helping you decide whether or not to get the COVID vaccine for your child?
    d) At what age would you feel most comfortable vaccinating your child against COVID-19? Why did you choose this age?
    e) Do you think that the COVID-19 vaccine is as safe as other/older vaccines and why do you think this?
    f) Have you talked to your child(ren) about getting the vaccine and if so, what was/were the conversation(s) like?

6. If the vaccine comes out tomorrow, how confident are you that you would be able to get the vaccine for your child?
    a) Where would you feel most comfortable getting the vaccine for your child?
        1. Doctor's office (PCP, pediatrician), drug store (Walgreens, CVS), school setting, drive up clinics
    b) What would be the easiest way or place for your child to get the vaccine?
    c) Who do you trust to get information regarding the COVID-19 vaccine for your child?
        1. If provider/health care professional not mentioned, ask why not.
    d) Are there any things that could make it easier for you to get the COVID-19 vaccines (on time) for your child?
        1. If not mentioned, ask about: Time off from work
        2. If not mentioned, ask about: Provider availability

3. If not mentioned, ask about: Location of clinics
4. If not mentioned, ask about: Support from your community
5. If not mentioned, ask about: Support from your family or friends

7. What benefits, if any, are there to your child receiving the COVID-19 vaccine?
   a) How effective do you think the vaccine is in preventing your child from getting covid? Tell me more about that.
   b) Would you feel that your child is safer if they received the vaccine? Tell me more about that.
   c) Do you believe that if you vaccinate your child, other members of your family are protected as well? Tell me more about that.
   d) How important (on a scale of 1-10, 1 being not at all important to 10 being very important) is it to you to vaccinate your child to protect those that cannot get vaccinated.

8. What, if any, concerns do you have about getting the vaccine for your child once it is approved?
   a) Who would you trust to talk through these concerns? (I.e., provider, partner, family member, friend, spiritual leader, government entities)
   b) What type of information or resources would be helpful to address these concerns?
   c) Would the concerns that you mentioned prevent you from vaccinating your child, once approved?
      1. How does your child's dislike of vaccines "pain" influence this decision?
   d) What can providers or public health officials do to increase your likelihood of vaccinating your child?

Closing

9. *[Provide a high-level summary of the interview, and ask the interviewee]* "Does that sound accurate? Is there anything you would like to add?"
10. Is there anything else about the COVID-19 vaccine that you would like to share?

Thank you for your time completing this interview. The information you provided is very valuable to us. To accurately ensure you get your gift card for participating, please confirm your email address is *[email address]*. If you feel you would like to contact anyone regarding this interview, the number to the Colorado Multiple Institutional Review Board is 303-724-1055. Thank you.
